# Ovarian volume as an independent marker for metabolic dysfunction in women with suspected androgen excess

**DOI:** 10.1016/j.xfre.2022.09.004

**Published:** 2022-09-26

**Authors:** Roy G. Handelsman, Sahar Wertheimer, Katherine VanHise, Rae A. Buttle, Ekaterina L. Clark, Erica T. Wang, Ricardo Azziz, Margareta D. Pisarska, Jessica L. Chan

**Affiliations:** a Cedars-Sinai Medical Center, Los Angeles, California; b University of Alabama at Birmingham, Birmingham, Alabama

**Keywords:** Ovarian volume, polycystic ovarian morphology, polycystic ovary syndrome, metabolic syndrome, androgen excess

## Abstract

**Objective:**

To determine whether ovarian volume (OV) alone is an independent marker for metabolic dysfunction in women with suspected androgen excess.

**Design:**

Retrospective cohort study.

**Setting:**

Tertiary academic reproductive endocrinology clinic.

**Patient(s):**

Women aged ≥21 years recruited/referred for symptoms related to androgen excess.

**Intervention(s):**

Transvaginal ovarian ultrasound, physical and medical evaluation, 2-hour 75-g oral glucose tolerance test (oGTT), and blood sampling.

**Main Outcome Measure(s):**

Prevalence of hyperandrogenism and metabolic dysfunction.

**Result(s):**

This study included 666 women, of whom 412 (61.9%) and 254 had OVs of >10 and ≤10 mL, respectively. An OV of >10 mL was associated with a higher prevalence of hirsutism (65.1% vs. 51.5%) than an OV of ≤10 mL. Polycystic ovary syndrome by the National Institutes of Health 1990 criteria was found in 67.3% and 51.4% of women with OVs of >10 and ≤10 mL, respectively. Metabolic parameters, including body mass index, waist circumference, and 1-hour insulin levels during the oGTT (odds ratio, 1.98; 95% confidence interval, 1.18–3.31), were significantly higher in women with an OV of >10 mL than in those with an OV of ≤10 mL. An OV of ≤10 mL had a 76.3% negative predictive value for hyperinsulinemia at 1 hour.

**Conclusion(s):**

In women with suspected androgen excess, an OV of >10 mL in at least 1 ovary is not associated with metabolic syndrome but is associated with younger age; an increased body mass index and waist circumference; a higher prevalence of hirsutism, oligoovulation, and polycystic ovary syndrome; and a higher 60-minute insulin level during the oGTT. Overall, an increased OV appears to be a good marker for hyperinsulinemia and hyperandrogenism in women suspected of having an androgen excess disorder.

**Discuss:** You can discuss this article with its authors and other readers at https://www.fertstertdialog.com/posts/xfre-d-22-00113

Polycystic ovary syndrome (PCOS) is a disorder characterized by hyperandrogenism, ovulatory dysfunction, and polycystic ovarian morphology (PCOM). Polycystic ovarian morphology is defined as an antral follicle count (AFC) of ≥12 or ovarian volume (OV) of >10 mL in at least 1 ovary (1). Although the criteria for diagnosis of PCOS has undergone significant discussion and modifications, including that of AFC to ≥20 per ovary, the definition of PCOM by OV has changed little (2). Obtaining an AFC requires full visualization of all antral (2–9 mm in diameter) follicles throughout an ovary, is generally considered to be more technically demanding than obtaining the OV, and is typically performed only in limited settings. Recent recommendations have also proposed that AFC be used only with newer technology ultrasounds (transducer frequency of ≥8 MHz) and to instead use OV in daily practice when this level of precision is not performed or available (3).

Despite the advantages of measuring OV rather than AFC, AFC has been studied more closely as a marker for metabolic dysfunction with limited predictability. In our study, the term “metabolic dysfunction” refers to insulin resistance (IR), hyperinsulinemia, or metabolic syndrome (MS) or any of the individual traits of MS, such as hyperglycemia, dyslipidemia, hypertension, or obesity. Women with PCOM defined by the accepted Rotterdam criteria at the time of study have been shown to have similar insulin, glucose, and lipid disturbances to those with PCOS, relative to reference subjects (4). Polycystic ovarian morphology on the basis of AFC is present in up to 32% of ovulatory women, and although associated with differences in androgens, body mass index (BMI), and waist circumference (WC), it has not been associated with more significant metabolic dysfunction (5, 6).

Alternatively, OV may be a more stringent marker for PCOM and metabolic dysfunction predictability because only 8.4% of ovulatory women meet the PCOS criteria on the basis of OV (5). One study demonstrated that in women with PCOS, OV was more closely associated with IR than AFC (7). Increased insulin levels have been directly linked to the production of androgen excess by the ovarian theca and stroma (8, 9). Consequently, this study aimed to determine whether OV alone is an independent marker for metabolic dysfunction in women with suspected androgen excess. We hypothesized that increased OV in this population is associated with greater androgenic and metabolic differences and that the sole finding of increased OV should prompt further evaluation.

## Materials and methods

### Subjects

This study included patients aged ≥21 years presenting for the evaluation of androgen excess to the Cedars-Sinai Medical Center reproductive endocrinology clinic from 2003–2010 who underwent transvaginal ultrasound assessment. The exclusion criteria were pregnancy and inability to provide informed consent. The study was approved by the institutional review board of Cedars-Sinai Medical Center. All subjects provided written informed consent before entry to a prospective study that sources the data on which this retrospective study was performed.

### Study Protocol

All subjects underwent physical examination. Data on age, height, and weight were obtained. The WC was measured at the narrowest portion of the torso approximately midway between the lower costal margin and iliac crest, and the hip circumference was measured over the widest portion of the gluteal and greater trochanteric region. The BMI and waist-to-hip ratio were then calculated. Clinical hyperandrogenism was assessed using the modified Ferriman-Gallwey (mFG) score (10, 11). Hirsutism was defined as having an mFG score of ≥4 (2). Menstrual history was obtained, and oligoovulation was defined as oligomenorrhea (≥35-day interval menstrual cycles or <10 bleeds per year) or by measuring the progesterone level between cycle days 20 and 24 in subjects claiming to be eumenorrheic (12).

All subjects underwent blood sampling. Fasting blood samples were obtained on days 3–8 of a spontaneous or progesterone-induced vaginal bleed (i.e., the follicular phase) for the measurement of the circulating total testosterone (T), free T, dehydroepiandrosterone sulfate (DHEA-S), and circulating insulin and glucose levels. Serum androgens were defined as a dichotomous variable and considered elevated on the basis of the laboratory’s reference range, when available. When reference ranges were unavailable or could not be validated, values were considered elevated if they exceeded the 95th percentile of all reported values. Other causes of hyperandrogenism, such as nonclassic congenital adrenal hyperplasia, were excluded, as previously reported (13).

The diagnosis of PCOS was made according to the National Institutes of Health (NIH) 1990 criteria (i.e., equivalent to phenotypes A and B of the Rotterdam criteria), including both the presence of oligoovulation and biochemical or clinical hyperandrogenism and excluding other known endocrinopathies, as previously described (14). The NIH 1990 criteria were selected to define PCOS because these define “classic” PCOS as having the strongest association with metabolic dysfunction (5, 15, 16, 17, 18, 19), a primary study end point, and do not include ovarian morphology, the primary variable being tested, in the definition.

All participants also underwent transvaginal ultrasonography using a 6.26-MHz transducer for AFC (counting all cystic areas 2–9 mm in diameter throughout the entirety of an ovary) and OV (using the ellipsoid approximation formula: 0.523 × length in cm × width in cm × thickness in cm). The vast majority of ultrasounds were performed by a single trained physician (M.P., details in the Acknowledgment section).

When data were available, the patients were assessed for the presence of MS using the definitions of the National Cholesterol Education Program Adult Treatment Panel III updated in 2005 by the American Heart Association and National Heart, Lung, and Blood Institute (20). Metabolic syndrome was diagnosed if ≥3 of the following 5 criteria were met: WC of ≥35 inches; hypertension (blood pressure of ≥130/85 mm Hg); fasting triglyceride (TG) level of ≥150 mg/dL; fasting high-density lipoprotein (HDL) cholesterol level of <50 mg/dL; and fasting blood glucose level of ≥100 mg/dL.

Subjects also underwent a standard 2-hour 75-g oral glucose tolerance test (oGTT), and the plasma insulin and glucose levels were determined at 0, 60, and 120 minutes. Glucose tolerance was determined on the basis of the American Diabetes Association criteria**,** with prediabetes defined as fasting plasma glucose levels of ≥100 but <126 mg/dL and/or 2-hour (120 minutes) glucose levels of ≥140 but <200 mg/dL. Type 2 diabetes mellitus was diagnosed by a basal glucose level of ≥126 mg/dL and a 2-hour glucose level of ≥200 mg/dL (21). The fasting glucose and insulin values were used to compute the homeostatic model assessment of IR (HOMA-IR) score. The HOMA-IR score was calculated using the following equation: (fasting glucose [mmol/L] × fasting insulin [mIU/L])/22.5. A severe HOMA-IR score was designated as a value >4, as previously described for identifying patients with IR (7, 22). However, these metabolic parameters were supplemental and, therefore, not collected on all patients.

### Hormonal Assays

The serum levels of total and free T were determined using liquid chromatography-tandem mass spectrometry (Quest Diagnostics; San Juan Capistrano, CA, or LabCorp; Calabasas, CA) and equilibrium dialysis (Quest Diagnostics or LabCorp), respectively (23). The DHEA-S level was determined using quantitative electrochemiluminescent immunoassay (ARUP Laboratories; Salt Lake City, UT, Quest Laboratories, or LabCorp). The glucose level during oGTT was determined using quantitative enzymatic assay, and the insulin level was determined using quantitative chemiluminescent assay (ARUP Laboratories). The levels of lipids, including total cholesterol, HDL, and TGs, were determined using quantitative chemiluminescent enzymatic assay (Diagnostic Products Corp., Los Angeles, CA). The low-density lipoprotein level was calculated using the Friedewald formula (24).

### Statistical Analysis

Women were classified into 2 groups according to OV: those with an OV of >10 mL (OV >10) in at least 1 ovary and those with an OV of ≤10 mL in both ovaries (OV ≤10). Categorical variables were compared using the χ^2^ test. Continuous variables were compared using Student’s *t* test or the Wilcoxon’s rank sum test, as appropriate. A *P* value of <.05 was considered significant. The mean AFC was determined to be the mean AFC for both ovaries. Similarly, the mean OV was determined to be the mean OV for both ovaries. Spearman’s rank correlation was calculated to correlate OV with AFC. Logistic regression was performed to adjust for age and BMI.

## Results

The study included 666 subjects aged ≥21 years with ovarian ultrasound data (Table 1). Of those, 412 (61.9%) had at least 1 ovary with an OV of >10 mL, and 254 (38.1%) had both ovaries with an OV of ≤10 mL. Women with an OV of ≤10 mL were older than those with an OV of >10 mL (31.9 ± 6.3 vs. 30.1 ± 5.6 years, respectively; *P*=.02). Women with an OV of ≤10 mL were less likely to be hirsute, with mean mFG scores of 4.5 and 6.3 in those with OVs of ≤10 and >10 mL, respectively, such that 51.5% of women with an OV of ≤10 mL were diagnosed with hirsutism vs. 65.1% of those with an OV of >10 mL. The 2 OV groups had a similar proportion of women with elevated total and free T levels; however, the proportion of women with an OV of ≤10 mL with elevated DHEA-S levels was higher than that of those with an OV of >10 mL (66.1% vs. 46.6%, respectively; *P* <.001).

**TABLE 1:** Basal and androgenic characteristics of the participants, stratified by ovarian volume.

Variable	OV of ≤10 mL	OV of >10 mL	*P* value^d^
n	254	412	
Age (SD), yrs	31.3 (6.3)	30.1 (5.6)	.02
Oligoovulatory, n (%) (N = 654)	142 (57.5)	298 (73.2)	<.001
Hirsute (mFG score of ≥4), n (%) (N = 640)	123 (51.5)	261 (65.1)	.001
mFG score (SD) (N = 640)	4.5 (4.3)	6.3 (5.3)	<.001
Elevated total T level, n (%) (N = 666)^a^	177 (69.7)	268 (65.1)	.22
Elevated free T level, n (%) (N = 666)^b^	180 (70.9)	266 (64.6)	.09
Elevated DHEA-S level, n (%) (N = 666)^c^	168 (66.1)	192 (46.6)	<.001
Clinical/biochemical HA, n (%) (N = 666)	219 (86.2)	366 (88.8)	.32
Mean OV (SD), mL (N = 666)	6.2 (1.9)	13.8 (5.6)	<.001
Mean AFC (SD) (N = 651)	10.5 (6.9)	16.0 (8.2)	<.001
PCOS by the NIH criteria, n (%) (N = 654)	127 (51.4)	274 (67.3)	<.001

*Note:* Each row designated with N to describe total study population with available data for each parameter. AFC = antral follicle count; DHEA-S = dehydroepiandrosterone sulfate; HA = hyperandrogenism; mFG = modified Ferriman-Gallwey; NIH = National Institutes of Health; OV = ovarian volume; PCOS = polycystic ovary syndrome; SD = standard deviation.

a The total T level was considered elevated when >45 ng/dL at Quest Diagnostics laboratories or >76 ng/dL at LabCorp, on the basis of the provided reference ranges. When the laboratory information was not available, the results were elevated if they exceeded the 95th percentile of the reported values.

b The free T level was considered elevated when >6.4 pg/mL at Quest Diagnostics laboratories or >2.2 pg/mL at LabCorp, on the basis of the provided reference ranges. When the laboratory information was not available, the results were elevated if they exceeded the 95th percentile of reported values.

c The DHEA-S level was considered elevated when >380 μg/dL at ARUP Laboratories, >325 μg/dL at Quest Diagnostics, or >270 μg/dL at LabCorp, on the basis of the provided reference ranges. When the laboratory information was not available, the results were elevated if they exceeded the 95th percentile of reported values.

d The *P* values of continuous variables were calculated using Student’s *t* test or the Wilcoxon rank sum test (depending on normality of data), and the χ^2^ test was used for categorical variables.

Anthropometrics were compared between the 2 groups (Table 2). An OV of >10 mL was associated with a significantly higher BMI (30.6 ± 8.0 vs. 28.3 ± 7.4 kg/m^2^, respectively; *P*=.0003), higher WC (98.4 ± 28.3 vs. 89.9 ± 18.9 cm, *P*<.001), and higher waist-to-hip ratio (0.86 ± 0.1 vs. 0.84 ± 0.1, *P*<.001). Other metabolic parameters, including hypertension, elevated TG level, low HDL level, and fasting blood glucose, were similar between the 2 groups. Overall, no significant difference was found for the presence of MS between the 2 groups. When comparing the glucose and insulin levels at times 0, 60, and 120 minutes during the oGTT (Table 2), all values were similar except for an elevated insulin level at 60 minutes (38.1% in the OV >10 group vs. 23.7% in the OV ≤10 group, *P*=.009; odds ratio [OR], 1.98).

**TABLE 2:** Metabolic characteristics of the participants, stratified by ovarian volume.

Variable	OV of ≤10 mL	OV of >10 mL	*P* value^a^
BMI (SD), kg/m^2^ (N = 653)	28.3 (7.4)	30.6 (8.0)	.0003
Waist-to-hip ratio (SD) (N = 507)	0.84 (0.1)	0.86 (0.1)	<.001
Waist circumference (SD), cm (N = 512)	89.9 (18.9)	98.4 (28.3)	<.001
HTN (BP of ≥130/85), n (%) (N = 525)	44 (23.2)	84 (25.1)	.62
Elevated TG (≥150), n (%) (N = 98)	5 (18.5)	17 (23.9)	.57
Low HDL (<50 mg/dL), n (%) (N = 97)	12 (44.4)	31 (44.3)	.99
Elevated fasting glucose level (≥100 mg/dL), n (%) (N=125)	5 (15.2)	11 (12.0)	.64
Metabolic syndrome, n (%) (N = 91)	6 (21.4)	18 (28.6)	.48
Elevated 0-min glucose level, n (%) (N = 326)	12 (10.2)	19 (9.1)	.76
Elevated 60-min glucose level, n (%) (N = 328)	15 (12.9)	27 (12.7)	.96
Elevated 120-min glucose level, n (%) (N = 333)	18 (15.1)	28 (13.1)	.61
Elevated 0-min insulin level, n (%) (N = 319)	15 (13.0)	42 (20.6)	.09
Elevated 60-min insulin level, n (%) (N=319)	27 (23.7)	78 (38.1)	.009
Elevated 120-min insulin level, n (%) (N = 195)	24 (46.2)	65 (45.5)	.93
Severe HOMA-IR (>4), n (%) (N = 352)	17 (15.0)	46 (23.2)	.08

*Note:* BMI = body mass index; BP = blood pressure; HDL = high-density lipoprotein; HOMA-IR = homeostatic model assessment of insulin resistance; HTN = hypertension; OV = ovarian volume; SD = standard deviation.

a The *P* values of continuous variables were calculated using Student’s *t* test or the Wilcoxon rank sum test (depending on normality of data), and the χ^2^ test was used for categorical variables

**FIGURE 1:**
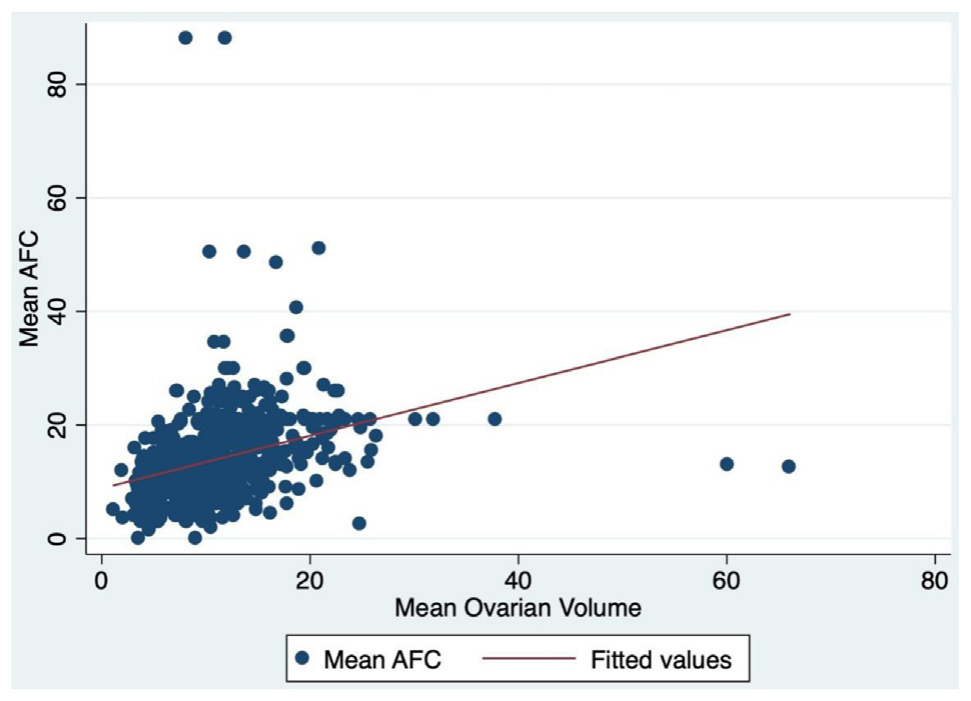
Correlation of the mean antral follicle count and mean ovarian volume. The fitted line (*r* = 0.51, *P*<.001) demonstrates that the mean ovarian volume is positively correlated with the mean antral follicle count. AFC = antral follicle count.

The mean OVs in women with OVs of ≤10 and >10 mL were 6.2 and 13.8 mL, respectively (*P*<.001). The mean ovarian AFCs for both groups were 10.5 and 16.0, respectively (*P*<.001). The mean OV was positively correlated with the mean AFC (*r* = 0.51) (Fig. 1). Women with an OV of >10 mL had a significantly higher prevalence of oligoovulation than those with an OV of ≤10 mL (73.2% vs. 57.5%, respectively; *P*<.001). The prevalence of PCOS by the NIH 1990 criteria was significantly higher in women with an OV of >10 mL (67.3%) than in those with an OV of ≤10 mL (51.4%, *P*<.001).

The ORs were calculated for oligoovulation, hirsutism, and elevated 60-minute postload insulin levels. The adjusted odds ratios (AORs) were also calculated, adjusting for BMI and age. An OV of >10 mL had higher odds of oligoovulation (OR, 2.02 [95% confidence interval {CI}, 1.45–2.82; *P*<.001]; AOR, 1.84 [95% CI, 1.3–2.60; *P*=.001]), hirsutism (OR, 1.75 [95% CI, 1.27–2.44; *P*=.001]; AOR, 1.43 [95% CI, 1.02–2.02; *P*=.04]), and elevated 60-minute postload insulin values (OR, 1.98 [95% CI, 1.18–3.31; *P*=.009]; AOR, 2.07 [95% CI, 1.16–3.68; *P*=.013]). An OV of >10 mL had lower odds of elevated DHEA-S levels (OR, 0.45 [95% CI, 0.32–0.62; *P*<.001]; AOR, 0.45 [95% CI, 0.33–0.63; *P*<.001]). It should be noted that the sample sizes were reduced for MS because the necessary diagnostic data were not collected in all subjects. Therefore, despite an overall population of 666 participants, the presence of MS could only be determined for 91 of these participants.

## Discussion

This study demonstrates that the sole finding of a higher OV is associated with the clinical markers of androgen excess and metabolic dysfunction. An OV of >10 mL was associated with a higher prevalence of hirsutism but not biochemical hyperandrogenemia. Moreover, an OV of >10 mL was associated with lower odds of elevated DHEA-S levels, suggesting that the source of androgen excess for this cohort was more likely ovarian in origin rather than adrenal. The origin of androgens may play a role in different clinical expressions of hyperandrogenism despite similar overall biochemical hyperandrogenemia. An OV of >10 mL was also associated with a higher BMI, WC, and 60-minute postload insulin levels. Even after adjusting for BMI and age, a higher OV was associated with higher odds of oligoovulation, hirsutism, and hyperinsulinemia.

A higher OV was associated with higher prevalence of PCOS by the NIH criteria. We specifically used the NIH criteria because it does not self-referentially include ovarian morphology in its diagnosis. Given the similar prevalence of hyperandrogenism, this demonstrates that the higher prevalence of PCOS in the larger-sized ovaries was based solely on the other component of the NIH criteria—oligoovulation.

Just as PCOS has known important clinical implications and associations with metabolic dysfunction, we explored the relationship of OV with metabolic parameters. Consistent with a previous study investigating PCOM (5), we found that a higher OV was associated with higher BMI and WC, which are both known to have significant effects on metabolic parameters and health. However, other important components of MS, including hypertension, fasting blood glucose, and low HDL levels, were similar between the groups. The composite (presence of MS) was, therefore, also similar between the 2 groups. These results are also consistent with previous findings (5). It should be noted that MS could only be evaluated in a limited sample of subjects. Although the prevalence of low HDL levels were similar between the 2 groups, both demonstrated a >40% prevalence of low HDL cholesterol levels, more than double the prevalence in women in the general population (25).

The prevalence of hyperinsulinemia is quite high in the overall cohort (13.0%–46.2%), demonstrating the importance of screening for insulin response during a glucose challenge in a population presenting for PCOS and androgen excess. Adams et al. (26) similarly noted an increased prevalence of hyperinsulinemia with PCOM. The finding that an OV of ≤10 mL has a 76.3% negative predictive value for hyperinsulinemia at 1 hour may allow for a higher threshold in testing for metabolic dysfunction in women with an OV of ≤10 mL. Although a study of 313 patients with Rotterdam-defined PCOS found an OV of >10 mL to be associated with higher fasting glucose levels and elevated HOMA-IR scores (7), our study did not demonstrate a difference in the fasting glucose levels between those women being evaluated for androgen excess with an OV of >10 or ≤10 mL and observed a nonsignificant trend toward elevated HOMA-IR scores. This may be because of the high overall prevalence of hyperinsulinemia in our population of patients with suspected androgen excess.

This study has several strengths, the largest being its sample size, including 666 well-phenotyped women, with sonographic and androgenic data. Although we were only able to assess for the presence of MS in approximately 14% our patients, we were able to gather other important metabolic data. This study also has limitations. Although the study results help comment on androgenic and metabolic factors associated with OV in women with suspected androgen excess, they may be less generalizable to an unselected population. Given the suggestion of greater ovarian secretion of androgens in this population because of decreased DHEA-S levels, further study in a patient population without baseline suspected androgen excess would be enlightening. It is possible that in a population without suspected androgen excess, the DHEA-S levels would be similar between the OV ≤10 and OV >10 cohorts; however, there would be more significant hyperandrogenism in the OV >10 cohort. Additionally, the mean BMI of our population was 29.7 kg/m^2^. Although other studies in the United States have suggested that obesity is highly prevalent in PCOS, this appears to primarily be from clinical referral bias in those studies (27, 28). Nonetheless, it is important to note the mean BMI of populations when attempting to compare results across studies.

Polycystic ovarian morphology is understood to be an important factor in the diagnosis and pathophysiology of PCOS. Classically, PCOM is classified by the OV and AFC. However, the AFC is relatively rarely measured outside of reproductive care clinics or the research setting. For greater generalizability, we sought to evaluate the performance of the more widely used OV as a single marker in the diagnosis of PCOS and its associated pathologies. Furthermore, the positive correlation between the OV and AFC (*r* = 0.51) in our study helps support the use of OV as a surrogate for AFC. We found that in women with suspected androgen excess, an OV of >10 mL in at least 1 ovary was associated with a higher prevalence of hirsutism, oligoovulation, PCOS by the NIH criteria, BMI, WC, and hyperinsulinemia than an OV of ≤10 mL. Thus, an increased OV appears to be a good marker for hyperinsulinemia and greater hyperandrogenism in women with suspected androgen excess disorder, and an OV of >10 mL suggests the need for a lower clinical threshold for testing for metabolic dysfunction.
